# Comparison of efficacy, safety, patients’ quality of life, and doctors’ occupational stress between lenalidomide‐based and bortezomib‐based induction in patients with newly diagnosed multiple myeloma

**DOI:** 10.1002/cam4.3762

**Published:** 2021-02-02

**Authors:** Limei Xu, Junru Liu, Beihui Huang, Lifen Kuang, Jingli Gu, Meilan Chen, Waiyi Zou, Juan Li

**Affiliations:** ^1^ Department of Hematology The First Affiliated Hospital of Sun Yat‐sen University Guangzhou China

**Keywords:** bortezomib, lenalidomide, occupational stress, quality of life, stem cell collection

## Abstract

**Background:**

In the new therapeutic era, comparisons between regimens containing lenalidomide and bortezomib are needed.

**Methods:**

In this single‐center, prospective study, patients received four to six cycles of lenalidomide+liposomal doxorubicin+dexamethasone (RAD) or bortezomib+liposomal doxorubicin+dexamethasone (PAD) every 4 weeks, with subsequent autologous stem cell transplantation (ASCT) and maintenance therapy. We compared the efficacy, safety, patients’ quality of life, and doctors’ occupational stress between RAD and PAD induction in newly diagnosed MM patients.

**Results:**

The complete response (CR) rate was comparable between the RAD and PAD groups after induction (30.8% vs. 32.0%, *p* = 0.92). Common adverse events, including infections, peripheral neuropathy, and gastrointestinal disturbances, were more frequent in the PAD group, while leukopenia and rashes were more common in the RAD group. Compared with PAD, RAD improved patients’ quality of life more quickly and caused less occupational stress for doctors. However, only 31.6% of patients collected adequate CD34+ cells (≥2 × 10^6^/kg) in the RAD group, which was significantly lower than that in the PAD group (95.5%, *p* < 0.001). The number of CD34+ cells collected was significantly higher in patients within three courses of RAD than in patients with four or five to six courses (14.18 ± 13.57 vs. 2.07 ± 2.42 vs. 1.51 ± 1.81 × 10^6^/kg, *p* = 0.028). The median progression‐free survival and overall survival of the two groups were not reached by the end of follow‐up.

**Conclusion:**

Compared to PAD, RAD induction had comparable efficacy and a significantly better safety profile, improved quality of life for patients, and reduced occupational stress for doctors. However, RAD induction may need to be limited to four cycles to avoid irreversible damage to hematopoietic stem cells.

**Clinical trial registration:**

This study was registered at www.chictr.org.cn (ChiCTR1900021558).

## Graphical abstract

Compared to PAD, RAD induction had a comparable efficacy and significantly better safety profile, improved quality of life for patients and reduced occupational stress for doctors. However, RAD had an effect on stem cell collection, which may need to be limited to 4 cycles to avoid irreversible damage to hematopoietic stem cells.

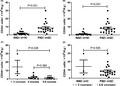

## 1 INTRODUCTION

Three‐drug combinations based on proteasome inhibitors and/or immunomodulators in combination with other drugs with different mechanisms are the preferred induction chemotherapy regimen for transplant‐eligible newly diagnosed multiple myeloma (NDMM) patients.^1^, ^2^ The combination of bortezomib, liposomal doxorubicin, and dexamethasone (PAD) has been widely used as the induction therapy for MM patients before transplantation.^3^ Although the use of bortezomib significantly improved the long‐term survival of patients with MM, the improvement in survival was accompanied by an increase in toxicity.^4^ A systematic review of phase III trials reported that the incidence of bortezomib‐related severe neuropathy (grades 3–4) ranged from 1% to 33.2% (median=8%).^5^ Chemotherapy‐induced peripheral neuropathy often has a major impact on the quality of life of patients, which then affects the compliance and efficacy of treatments.^6^ A meta‐analysis showed that lenalidomide did not significantly increase the risk of peripheral neuropathy compared with bortezomib.^7^

With the continuous emergence of new drugs, the optimal induction regimen for NDMM patients should be well balanced in efficacy, safety, and cost. Regimens containing lenalidomide, such as lenalidomide, liposomal doxorubicin, and dexamethasone (RAD), were reported to be safe and effective for MM in a few clinical trials. Baz et al.^8^ reported that the RAD regimen resulted in a 77.2% overall response rate (ORR) in NDMM patients. In addition, Terpos et al.^9^ found that the ORR was 66.7% after four cycles of RAD induction. However, all of these trials were single‐arm phase II trials, and the regimens were not compared with other induction regimens. Comparisons between regimens containing lenalidomide and bortezomib are needed.

Therefore, we conducted a prospective clinical trial of RAD induction sequentially by autologous stem cell transplantation (ASCT) and maintenance therapy to treat NDMM patients and compared the efficacy, safety, and effects on subsequent stem cell collection and hematopoietic reconstitution, patients’ quality of life, and doctors’ occupational stress between RAD and PAD induction in these patients.

## 2 METHODS

### 2.1 Patients

Eligible MM patients who were previously untreated met the diagnosis of MM according to International Myeloma Working Group (IMWG) criteria.^10^ The patients were between 18 and 70 years old and were suitable for ASCT. The main exclusion criterion was a creatinine clearance rate <60 ml/min. All of the patients provided written informed consent.

### 2.2 Study design

This study was a single‐center, prospective, open‐label, nonrandomized, comparative, phase II clinical trial that was approved by the Clinical Research and Animal Ethics Committee of our hospital. This study was registered at the Chinese Clinical Trial Registry.

A total of 60 NDMM patients were enrolled from November 2017 to September 2018 and were divided into the RAD group (*n* = 30) and the PAD group (*n* = 30) according to their wishes after we fully communicated with them the benefits and risks of their disease and treatment choices. Since this study was nonrandomized, we first used propensity score matching (PSM) to minimize bias between the two groups. The factors that may affect the therapeutic effect or survival prognosis of the patients were included, including their age (≤60 or >60), International Staging System (ISS) stage (I, II, III), cytogenetics (high risk or standard risk; briefly, translocation 4;14 [t (4;14)] and/or del[17p] and/or t [14;16] determined by FISH were defined as high‐risk cytogenetics; not carrying these mutations was defined as standard risk cytogenetics), and lactate dehydrogenase (LDH) levels (≥240 or <240 U/L). First, 30 patients in the RAD group and 35 patients in the PAD group were selected and matched according to their age, ISS stage, cytogenetics, and LDH levels using a 1:1 nearest neighbor matching algorithm (calipers of a width equal to 0.08). Finally, a total of 60 patients were enrolled, including 30 patients in the RAD group and 30 patients in the PAD group. To further ensure the similarities between the two groups, we compared the baseline clinical data of the 60 patients. There was no significant difference in age, sex, hemoglobin level, serum creatinine level, serum calcium level, LDH level, M‐protein type, ISS stage, or cytogenetics between the two groups, which further confirmed the comparability between the two groups.

### 2.3 Treatments

The patients received four to six cycles of RAD (R, 25 mg, po, d1‐21; A, 40 mg/m^2^, IV drip, d1‐2; and D, 20 mg, IV drip, d1‐4) or PAD (P, 1.3 mg/m^2^, iv, d1, 4, 8, 11; A and D, every 4 weeks) with subsequent ASCT and maintenance therapy. The mobilizing regimen was cyclophosphamide (CTX 3 g/m^2^, IV drip d1) + granulocyte colony‐stimulating factor (G‐CSF, 300 μg, ih QD from d2) or G‐CSF alone (G‐CSF, 300 μg, ih QD d1‐5). The maintenance regimen was thalidomide (200 mg QN). Patients who changed the established treatment plan for any reason (excluding disease progression) or were lost to follow‐up before reaching the end point (disease progression or death) were defined as discontinuation/interruption. Common reasons for discontinuation/interruption include adverse events, mobilize failure, poor efficacy, or voluntary withdrawal.

### 2.4 Response and safety evaluation

The efficacy was evaluated according to the IMWG 2016 efficacy evaluation criteria.^11^ The efficacy of complete response (CR), very good partial response (VGPR), and partial response (PR) was analyzed. In addition, we also used minimal residual disease (MRD) to evaluate the efficacy for these patients. The sensitivity of our flow cytometry detection for MRD was 10^−5^.

Progression‐free survival (PFS) was calculated from the start of treatment to disease progression, death, or the last follow‐up, and overall survival (OS) was calculated from the start of treatment until death or the last follow‐up.

The intensity of adverse events (AEs) was graded and recorded according to the common toxicity standard grading system (National Cancer Institute [NCI] Common Terminology Criteria for Adverse Events [CTCAE], version 5.0).

### 2.5 Quality of life and occupational stress assessments

The Quality of Life Questionnaire (QLQ)‐C30^12^ and myeloma‐specific module 20 (MY20)^13^ were used to evaluate patients’ quality of life. A questionnaire on effort‐reward imbalance was used to evaluate doctors’ and nurses’ occupational stress.^14^ For specific information on the questionnaire, see the Supplementary Material.

### 2.6 Statistical methods

The results reported were as of April 2020, with a median follow‐up of 23.97 (17.07–29.40) months from the start of treatment. SPSS Statistics software, version 23 (Chicago, IL, USA), was utilized for the statistical analysis. We analyzed the numerical variables by Student's *t*‐test or the Mann–Whitney U test. For the comparison of categorical variables, we used the Chi‐square test or Fisher's exact test. The Kaplan–Meier method was used for survival analysis, and differences were analyzed using the log‐rank test. *p* < 0.05 was considered to be statistically significant.

## 3 RESULTS

### 3.1 Basic characteristics of the patients in the RAD and PAD groups

The demographic and clinical characteristics of all of the enrolled patients are listed in Table 1. The baseline characteristics were similar between the RAD and PAD groups. In terms of M‐protein types, the IgG type was dominant in both groups. In addition, the ISS stage of both groups was mainly stage II. There was also a balanced distribution between the two groups in terms of cytogenetics. A consort diagram of the patients throughout the study is shown in Figure S1.

**TABLE 1:** Characteristics of the patients in this study

Characteristics	RAD (N = 30)	PAD (N = 30)	*P*
Mean age, year, (± SD)	56 ± 9.19	54 ± 7.81	0.470
Sex ratio, male/female (number)	15/15	17/13	0.605
Mean serum hemoglobin, g/L, (± SD)	100 ± 19.46	103 ± 20.66	0.490
Mean serum creatinine, µmol/L, (± SD)	77 ± 19.46	77 ± 22.47	0.990
Mean serum calcium, mmol/L, (± SD)	2.39 ± 0.36	2.38 ± 0.29	0.906
Mean serum LDH level, U/L, (± SD)	168 ± 61.59	178 ± 86.17	0.587
Type of myeloma			0.961
IgG	18 (60.0%)	16 (53.3%)	
IgA	7 (23.3%)	8 (26.7%)	
IgD	1 (3.3%)	1 (3.3%)	
Light chains only	4 (13.4%)	5 (16.7%)	
ISS stage			0.806
Ⅰ	7 (23.3%)	8 (26.7%)	
Ⅱ	16 (53.4%)	17 (56.7%)	
Ⅲ	7 (23.3%)	5 (16.6%)	
Cytogenetic abnormality by FISH			0.117
17p‐	1 (3.3%)	3 (10.0%)	
t (4;14)	5 (16.7%)	2(6.67%)	
t (14;16)	0	1 (3.3%)	
t (11;14)	0	3 (10.0%)	
13q‐	10 (33.3%)	7 (23.3%)	
1q21+	10 (33.3%)	7 (23.3%)	

Abbreviations: FISH, fluorescence in situ hybridization; ISS, international staging system; LDH, lactate dehydrogenase.

### 3.2 Response

The ORR and CR rates were comparable between the RAD and PAD groups after induction (92.3% vs. 80.0%, *p* = 0.20; and 30.8% vs. 32.0%, *p* = 0.92, respectively, Table 2). Similarly, we observed no statistically significant differences in the ORR or CR rates between the RAD and PAD groups at 3, 6, and 9 months after transplantation. In addition, there were also no statistically significant differences in the negative rate of MRD between the two groups at the end of induction therapy or at 3, 6, and 9 months after ASCT (Table 2). The responses and MRD negative status post induction and after ASCT reported at different time points are cumulatively calculated.

**TABLE 2:** Treatment response during induction and after ASCT

	RAD *N* (%)	PAD *N* (%)	*P*
Response after induction (2 cycles)	*N* = 29	*N* = 30	
ORR (PR or better)	21 (72.0)	19 (63.3)	0.460
CR	2 (6.9)	5 (16.7)	0.450
Response after induction (4 cycles)	*N* = 26	*N* = 25	
ORR (PR or better)	24 (92.3)	20 (80.0)	0.200
CR	6 (23.1)	8 (32.0)	0.480
Response after induction (4–6 cycles)	*N* = 26	*N* = 25	
ORR (PR or better)	24 (92.3)	20 (80.0)	0.200
CR	8 (30.8)	8 (32.0)	0.920
Response after ASCT (3 months)	*N* = 13	*N* = 21	
ORR (PR or better)	12 (92.3)	20 (95.2)	0.724
CR	7 (53.9)	11 (52.4)	0.934
Response after ASCT (6 months)	*N* = 12	*N* = 20	
ORR (PR or better)	10 (83.3)	19 (95.0)	0.639
CR	7 (58.3)	13 (65.0)	0.706
Response after ASCT (9 months)	*N* = 11	*N* = 19	
ORR (PR or better)	9 (81.8)	18 (94.7)	0.613
CR	6 (54.6)	15 (79.0)	0.321
MRD negative status (10^−5^)			
after induction (4–6 cycles)	5 (19.2)	7 (28.0)	0.560
3 months after ASCT	5 (38.5)	9 (42.9)	0.800
6 months after ASCT	5 (41.7)	11(55.0)	0.465
9 months after ASCT	5 (45.5)	12 (63.2)	0.575

Abbreviations: ASCT autologous stem cell transplantation; CR complete response; MRD minimal residual disease; ORR overall response rate; PR partial response.

We conducted a subgroup analysis of the CR rate at the end of induction chemotherapy in the two groups (Table S1). We defined the patients whose prognosis was classified as R‐ISS Ⅰ as the low‐risk group and those with prognoses of R‐ISS Ⅱ/Ⅲ as the high‐risk group.^15^ The results showed that in the RAD group, the CR rate in the low‐risk group was significantly greater than that in the high‐risk group (83.3% vs. 15.0%, *p* = 0.007). However, in the PAD group, there was no significant difference in the CR rate between the low‐risk and high‐risk groups.

### 3.3 Safety

We analyzed the treatment‐emergent adverse events (TEAEs) of 60 patients during induction chemotherapy (Table 3). Overall, hematological toxicities, such as leukopenia, were more common in the RAD group than in the PAD group (80.0% vs. 53.3%, *p* = 0.028). In terms of nonhematological AEs, the proportions of patients with febrile symptoms and pulmonary infection in the RAD group were significantly lower than those in the PAD group (10.0% vs. 53.3%, *p* = 0.001; and 40.0% vs. 66.7%, *p* = 0.038, respectively). The proportion of patients with grade 3 diarrhea in the RAD group was also lower than that in the PAD group (13.3% vs. 40.0%, *p* = 0.020). In addition, the incidence of rash in the RAD group was significantly higher than that in the PAD group during induction therapy (33.3% vs. 6.7%, *p* = 0.01), while the proportion of patients with peripheral neuropathy in the RAD group during induction therapy was significantly lower than that in the PAD group (10.0% vs. 46.7%, *p* = 0.001).

**TABLE 3:** Most common adverse events during introduction

	Any grade N (%)			Grade 3 or 4 N (%)		
	RAD (*N* = 30)	PAD (*N* = 30)	*P*	RAD (*N* = 30)	PAD (*N* = 30)	*P*
Hematological adverse events						
Leukocytopenia	24(80.0)	16(53.3)	0.028	5(16.7)	1(3.3)	0.197
Anemia	10(33.3)	8(26.7)	0.778	0	0	/
Thrombocytopenia	4(13.3)	10(33.3)	0.067	1(3.3)	2(6.7)	0.554
Nonhematological adverse events						
Infection						
Fever	3(10.0)	16(53.3)	0.001	0	0	/
Lung infection	12(40.0)	20(66.7)	0.038	1(3.3)	2(6.7)	0.554
Herpes zoster	2(6.7)	7(23.3)	0.148	0	4(13.3)	0.121
Gastrointestinal						
Diarrhea	8(26.7)	18(60.0)	0.001	4(13.3)	12(40.0)	0.020
Constipation	11(36.7)	13(43.3)	0.598	0	0	/
Nausea and vomiting	3(10.0)	8(26.7)	0.095	0	0	/
Pancreatitis	0	1(3.3)	1	0	1(3.3)	1
Dermatologic						
Rash	10(33.3)	2(6.7)	0.01	2(6.7)	0	0.472
Swelling/erythema	0	0	/	0	0	/
Neurologic						
Peripheral neuropathy	3(10.0)	14(46.7)	0.001	0	4(13.3)	0.121
Cardiovascular						
Arrhythmia	1(3.3)	1(3.3)	1	1(3.3)	0	1
Others						
Pulmonary embolism	0	0	/	0	0	/
Vein thrombosis	0	0	/	0	0	/
Edema	6(20.0)	2(6.7)	0.255	0	0	/
Fatigue	12(40.0)	12(40.0)	1	0	0	/
Dizziness	6(20.0)	5(16.7)	0.739	0	0	/

/, not calculated.

Both groups of patients were treated with thalidomide (200 mg QN) after transplantation. Among those 34 patients, three (8.8%) developed grades 1–2 rash, five (14.7%) developed grades 1–2 edema, and two (5.9%) developed new grades 1–2 peripheral neuropathy. There were two (5.9%) patients with a reduction in thalidomide (150 mg QN) because of adverse events. By the deadline of follow‐up, no patient withdrew from treatment due to the adverse events of thalidomide.

### 3.4 Stem cell collection

**FIGURE 1:**
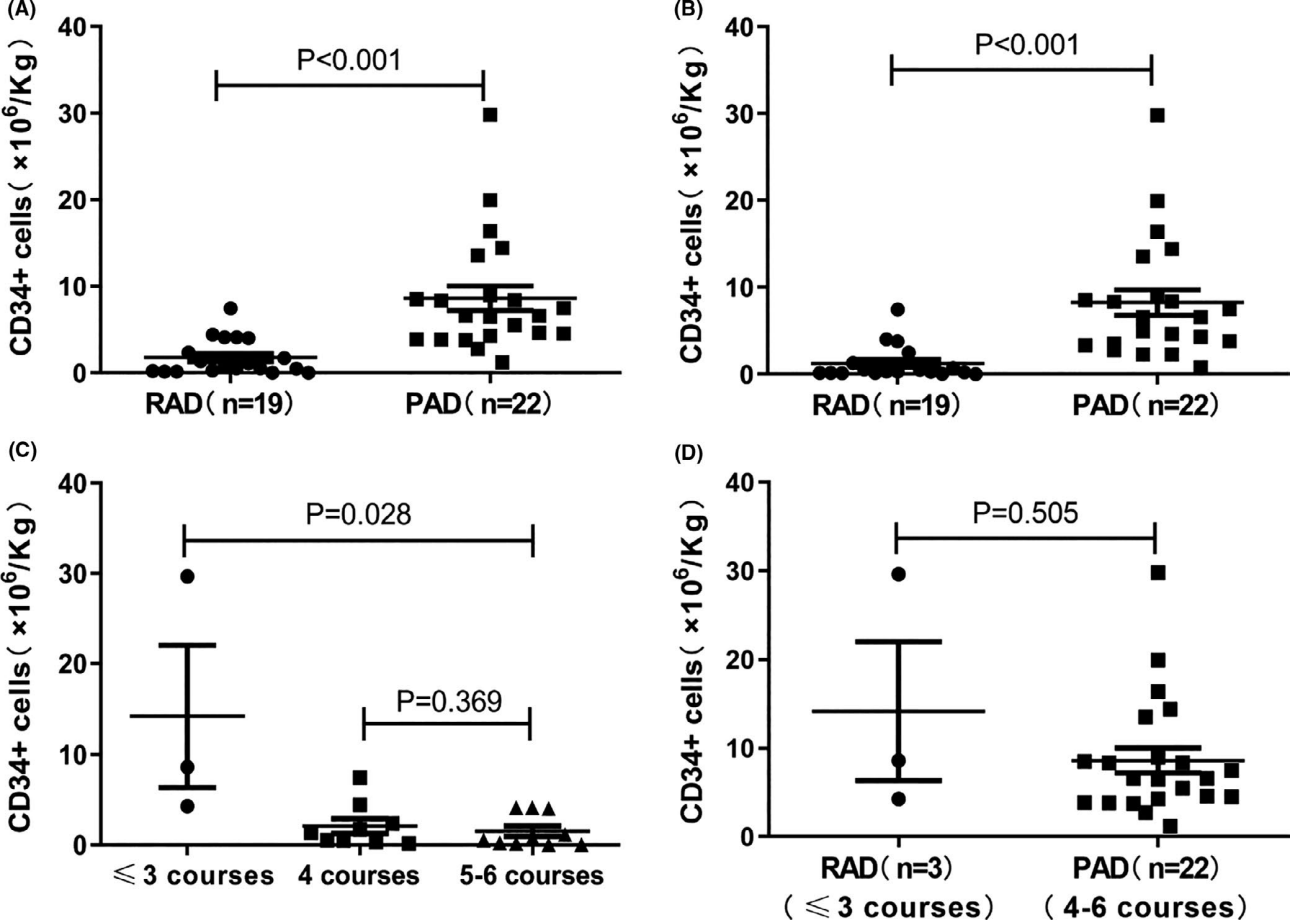
The effects of RAD and PAD induction on stem cell collection in MM patients. (A) Comparison of the total number of CD34+ cells collected from the RAD and PAD groups. (B) Two days after stem cell collection, the numbers of CD34+ cells collected from the RAD and PAD groups were compared. (C) The number of CD34+ cells collected among different courses of RAD induction. (D) Comparison of the number of CD34+ cells collected from RAD induction for ≤3 courses and PAD induction for 4–6 courses

In the RAD group, 19 patients had completed stem cell mobilization, of whom 17 patients were mobilized with the CTX+G‐CSF regimen, and two patients were mobilized with G‐CSF alone. In the PAD group, 22 patients completed stem cell mobilization, of whom 21 patients were mobilized with the CTX+G‐CSF regimen and one patient with G‐CSF alone. Our results showed that there was no significant difference in the collection of total nucleated cells (TNCs) between the RAD group and the PAD group (8.32 ± 6.14 × 10^8^/kg vs. 7.45 ± 2.92 × 10^8^/kg, *p* = 0.661), but the mean number of CD34^+^ cells collected in the RAD group was significantly lower than that in the PAD group (1.77 ± 2.08 × 10^6^/kg vs. 8.63 ± 6.64 × 10^6^/kg, *p* < 0.001; Figure 1A), regardless of whether CTX+G‐CSF or G‐CSF alone was used for mobilization (Table 4). Similarly, the proportions of CD34^+^ cells ≥2 or 4 × 10^6^/kg collected in the RAD group were also lower than those in the PAD group (Table 4).

**TABLE 4:** Stem cell mobilization, transplantation, and hematopoietic reconstruction

	RAD	PAD	*P*
Stem cell mobilization			
Mean number of CD34+ cells collected mobilized with CTX+G‐CSF (×10^6^/kg)	1.88 ± 2.18	8.83 ± 6.74	<0.001
Mean number of CD34+ cells collected mobilized with G‐CSF alone (×10^6^/kg)	0.91	4.29	0.667
Proportion of CD34+ cells≥2 × 10^6^/kg, n (%)	6 (31.6)	21 (95.5)	<0.001
Proportion of CD34+ cells≥4 × 10^6^/kg, n (%)	5 (26.3)	17 (77.3)	0.001
Number of days needed to collect CD34+ cells ≥1 × 10^6^/kg, d (range)	4 (1–8)	1 (1–3)	<0.001
Stem cell transplantation			
Proportion of patients undergoing ASCT after stem cell mobilization, n (%)	13 (64.8)	22 (100.0)	0.016
Reinfusion numbers of CD34+ cells, median (range, ×10^6^/kg)	1.83 (0.79–2.94)	3.73 (1.22–15.3)	<0.001
Hematopoietic reconstruction			
Median time to recovery of ANC >0.5 × 10^9^/L, d (range)	10 (9–12)	9 (9–11)	0.083
Median time to recovery of PLT >20 × 10^9^/L, d (range)	12 (9–15)	11 (9–16)	0.023

Abbreviations: ANC, absolute neutrophil count; ASCT, autologous stem cell transplantation; PLT, platelets.

In addition, we also analyzed the number of days for collecting stem cells. The number of days needed to collect CD34+ cells ≥1 × 10^6^/kg was greater in the RAD group (median days: 4; range: 1–8 days) than in the PAD group (median days: 1; range: 1–3 days; *p* < 0.001; Table 4). For patients with more than 4 days of stem cell collection, the number of collection days was calculated as the sum of two mobilization days. In addition, 2 days after stem cell collection, the number of CD34+ cells collected in the RAD group was also significantly lower than that in the PAD group (1.26 ± 1.93 × 10^6^/kg vs. 8.25 ± 6.91 × 10^6^/kg, *p* < 0.001; Figure 1B).

We further analyzed the collection of CD34+ cells in patients with different courses of treatment (Figure 1C). The results showed that the number of CD34+ cells collected from patients within three courses of RAD was significantly greater than that collected from patients with four or five to six courses (14.18 ± 13.57 vs. 2.07 ± 2.42 vs. 1.51 ± 1.81 × 10^6^/kg, *p* = 0.028). In addition, there was no significant difference in the number of CD34+ cells collected between the RAD regimen in ≤3 courses and the PAD regimen in four to six courses (14.18 ± 13.57 vs. 8.63 ± 6.64 × 10^6^/kg, *p* = 0.505; Figure 1D).

### 3.5 Transplantation and hematopoietic reconstruction

Stem cell mobilization was performed in 19 patients in the RAD group, but due to the failure of mobilization in some patients, only 13 patients (68.4%) underwent ASCT, while 22 patients in the PAD group underwent stem cell mobilization, and all of them completed ASCT. We also observed the hematopoietic reconstruction time after ASCT in both groups. The median time to recovery of platelets (PLT) >20 × 10^9^/L in the RAD group was longer than that in the PAD group (12 days vs. 11 days, *p* = 0.023). We further calculated that the median reinfusion numbers of CD34+ cells in the RAD and PAD groups were 1.83 × 10^6^/kg and 3.73 × 10^6^/kg, respectively (*p* < 0.001). Therefore, the prolongation of hematopoietic reconstitution time in the RAD group was mainly related to the insufficient number of reinfusion stem cells (Table 4).

### 3.6 Survival analyses

**FIGURE 2:**
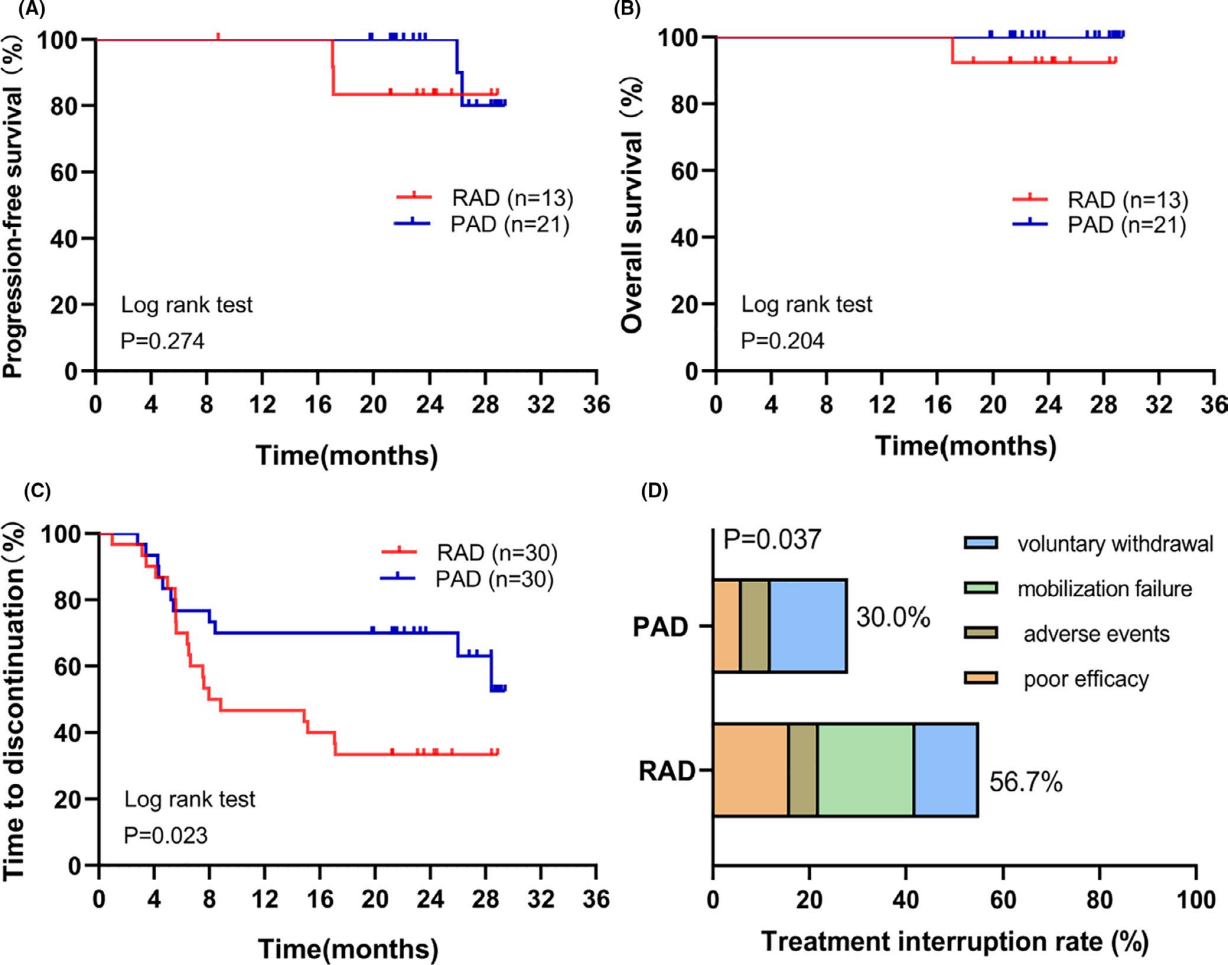
Comparison of treatment interruption and survival prognosis between the RAD and PAD groups. (A) The PFS of patients undergoing transplantation and maintenance therapy in the two groups. (B) The OS of patients undergoing transplantation and maintenance therapy in the two groups. (C) Kaplan–Meier plots of the time‐to‐study discontinuation between the RAD and PAD groups. (D) Comparison of the interruption rate between the RAD and PAD groups

We analyzed the survival of patients who received four to six cycles of RAD or PAD every 4 weeks, with subsequent ASCT and maintenance therapy. The median follow‐up time was 23.97 (17.07–29.40) months by April 2020, and the median PFS and OS of the two groups were not reached (Figure 2A and B). Further follow‐up is needed. The 12‐month PFS and OS rates in both groups were 100%. In the RAD group, the 18‐month PFS rate was 84.62%, and the OS rate was 92.31%, whereas in the PAD group, the 18‐month PFS and OS rates were 100%.

The median time to discontinuation of patients in the RAD group was 7.97 months, while it was not reached in the PAD group (*p* = 0.017, Figure 2C). In addition, during the whole treatment, the interruption rate in the RAD group was 56.7%, which was significantly greater than that in the PAD group (30.0%, *p* = 0.037). Twenty percent of the patients in the RAD group dropped out because of mobilization failures and failed to perform ASCT, which was the main reason for interruption (Figure 2D).

### 3.7 Health‐related quality of life

**FIGURE 3:**
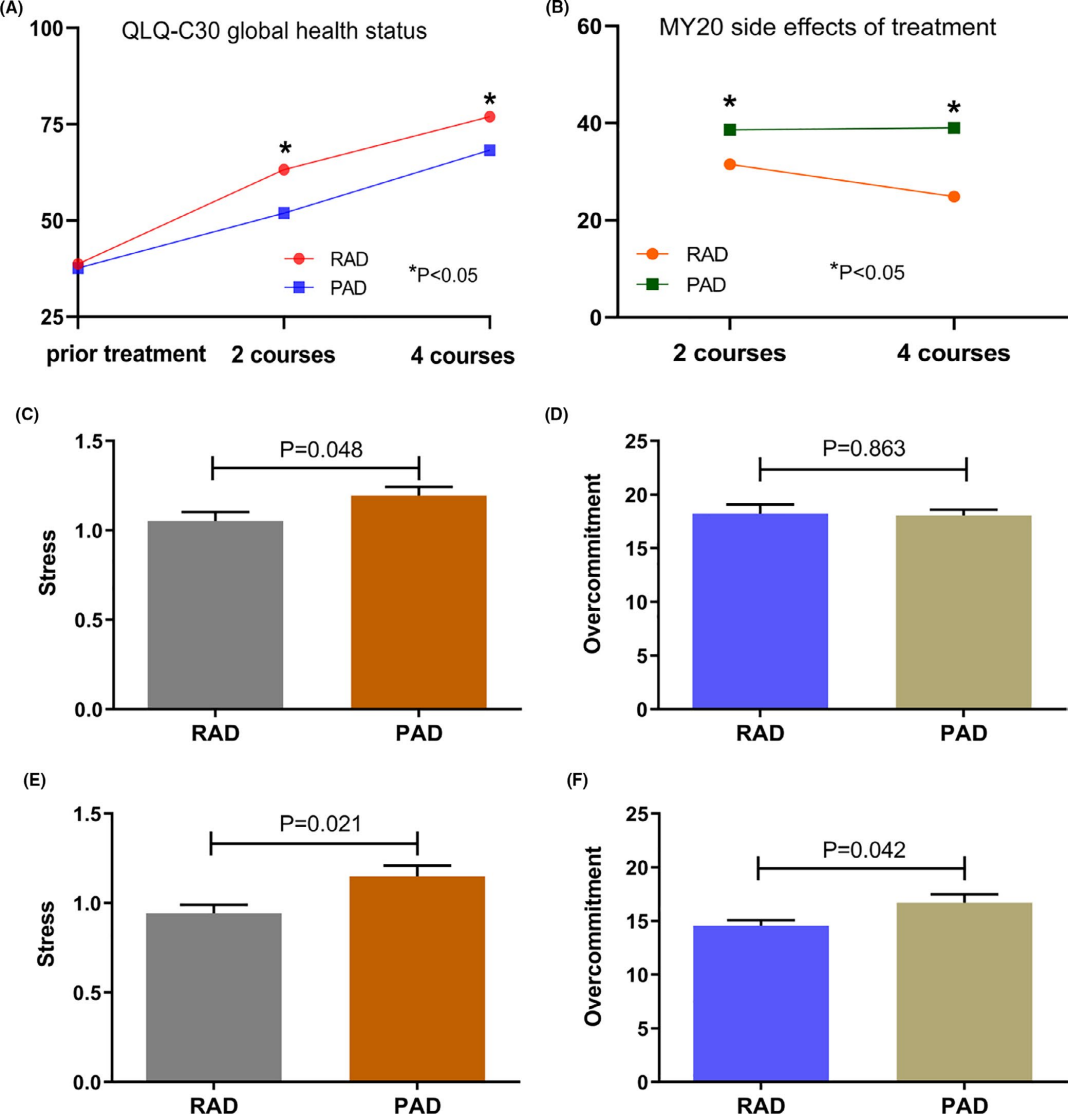
The impact of RAD and PAD on the quality of life of patients and the occupational stress of doctors and nurses. (A) On the QLQ‐C30 global health status scale, the impact of RAD and PAD on the quality of life of patients after two courses and four courses of induction therapy. (B) On the MY20 disease symptoms scale, the impact of RAD and PAD on the quality of life of patients after two courses and four courses of induction therapy. (C) Comparison of the impact of RAD and PAD on the occupational stress of doctors. (D) Comparison of doctors’ overcommitment to patients undergoing the RAD and PAD regimens. (E) Comparison of the impact of RAD and PAD on the occupational stress of nurses. (F) Comparison of nurses’ overcommitment to patients undergoing the RAD and PAD regimens

We found that, with increasing courses of treatment, both RAD and PAD induction therapy can improve the quality of life of MM patients (Tables S2 and S3). We performed a comparative analysis of the patient questionnaire responses collected from the RAD group and PAD group after performing integral statistics in different dimensions. The results of the RAD group were better than those of the PAD group after two courses or four courses of treatment (63.19 ± 3.68 vs. 51.89 ± 3.05, *p* = 0.024; and 76.98 ± 2.43 vs. 68.25 ± 3.52, *p* = 0.048, respectively; Figure 3A). In addition, in the dimension of the side effects of treatment on the MY20 questionnaire, the scores of the RAD group in two courses or four courses of treatment (31.50 ± 2.72 vs. 38.63 ± 1.84, *p* = 0.039; 24.87 ± 3.99 vs. 39.02 ± 2.52, *p* = 0.005, respectively; Figure 3B) were lower than those of the PAD group, suggesting that the side effects of the RAD regimen were fewer than those of the PAD regimen and had less of an effect on patients’ quality of life.

### 3.8 Occupational stress assessment for doctors and nurses

The use of different induction schemes not only affects patients but also exerts different work pressure on doctors and nurses. We collected valid questionnaires from 40 doctors and 14 nurses. The results showed that, during the induction treatment, doctors had more occupational stress treating patients using the PAD regimen than treating patients using the RAD regimen (1.19 ± 0.19 vs. 1.05 ± 0.17, *p* = 0.048; Figure 3C), but there was no significant difference in overcommitment (intrinsic input) between the two groups (18.03 ± 3.13 vs. 18.05 ± 2.24, *p* = 0.863; Figure 3D). Similarly, compared with patients using the RAD regimen, nurses felt more pressure with patients using the PAD regimen (1.15 ± 0.06 vs. 0.95 ± 0.05, *p* = 0.021; Figure 3E), as well as more overcommitment (16.71 ± 0.78 vs. 14.57 ± 0.53, *p* = 0.042; Figure 3F). Doctors and nurses were less stressed when RAD induction was used on patients, which might be related to less worry about drug side effects, shorter average hospital stays, and less time spent recording and processing (Figure S2).

## 4 DISCUSSION

RAD was first used for relapsed and refractory MM patients and then gradually used in NDMM patients as an induction regimen, but there have been few reports. Our results showed that at the end of induction, the ORR in the RAD group was 92.3%, which was comparable to that in the PAD group (80.0%, *p* = 0.20). In previous studies, after RAD induction, the ORR was 66–77%,^8^, ^9^ indicating that RAD as an induction regimen has a good effect. Although the CR rate after induction between the RAD and PAD groups was comparable (30.8% vs. 32.0%, *p* = 0.92), the CR rate after two courses of RAD induction was significantly lower than that in the PAD group (6.9% vs. 16.8%, *p* = 0.45), indicating that RAD induction was slow to take effect. In addition, stratified analysis showed that the CR rate in patients with RAD induction was significantly lower in the high‐risk group than in the low‐risk group (15.0% vs. 83.3%, *p* = 0.007), suggesting that RAD could not overcome the poor prognostic factors of MM. Similarly, some clinical trials have shown that bortezomib treatment appears to improve the CR rate in t[4;14] and del[17p] MM patients.^16^, ^17^ However, it has not been reported that lenalidomide can overcome the effect of adverse prognostic factors on the CR rate in MM patients. In addition, we also evaluated the ORR, CR rate, and MRD negative rate within 9 months after transplantation. There was no significant difference between the two groups, which may be related to the short follow‐up time. The median follow‐up time was 23.97 (17.07–29.40) months, and the median PFS and OS of the two groups were not reached. Thus, we still need to continue follow‐up to further evaluate the effects of different treatment regimens on the long‐term efficacy of the two groups.

Overall, the incidence of leukopenia was higher in the RAD group (80.0%) than in the PAD group (53.3%), but the higher percentage of leukopenia induced by RAD did not increase the risk of infection compared with that in the PAD group. Our results showed that the incidence of infection‐related AEs, including fever, pulmonary infection, and herpes zoster, in the RAD group was lower than that in the PAD group. Some studies have reported that bortezomib can act on T lymphocytes, lead to a decrease in CD4+ T lymphocytes, and suppress Th17 differentiation in human naive T cells in culture,^18^, ^19^ which could be the reason for the increased risk of infection. In addition, our results also showed that the incidence of rash induced by RAD during induction was higher than that in the PAD group (33.3% vs. 6.7%, *p* = 0.01), considering that lenalidomide was associated with an increased risk of rash. A meta‐analysis including 737 MM patients who were treated with a lenalidomide regimen showed that the overall incidence of all‐grade and ≥3‐grade rash was 27.2% and 3.6%, respectively,^20^ which was similar to our results. However, the incidence of diarrhea and peripheral neuropathy in the RAD group was lower than that in the PAD group, and we found that the incidence of peripheral neuropathy in the PAD group significantly increased with the increase in the number of treatment courses, which was related to the cumulative dose of bortezomib‐related peripheral neuropathy.^21^

In the new therapeutic era, optimizing the health‐related quality of life of MM patients has become an important treatment goal. There have been a number of clinical trials comparing the effects of drugs on the quality of life of MM patients.^22^, ^23^, ^24^ Our results showed that the overall health status of the RAD group improved more significantly, which might be related to the relatively convenient oral administration of lenalidomide and fewer treatment‐related side effects. In addition, to better balance the occupational stress of medical staff, we also analyzed the occupational stress and intrinsic input caused by different treatment schemes for patients. The results showed that the occupational stress caused by the PAD regimen was greater, which might be related to doctors being more worried about adverse drug reactions or extended hospital stays when patients used the PAD regimen.

In terms of stem cell collection, our results showed that the collection of CD34+ cells in MM patients induced by RAD was significantly lower than that in MM patients induced by PAD, suggesting that lenalidomide has a significant effect on stem cell collection. The high failure rate of stem cell mobilization in the RAD group also led to difficulty with ASCT and hematopoietic reconstitution, which was the major reason for treatment interruption. However, in the study by Terpos et al.,^9^ 89% of patients successfully mobilized stem cells after four courses of RAD induction, and the average number of CD34+ cells collected was 8.94 × 10^6^/kg. In addition, Baz et al.^8^ reported that in 31 patients with MM induced by the RAD regimen for four to eight courses, the number of stem cells collected from all of the patients was ≥2 × 10^6^/kg. Compared with the above study, the dosage of lenalidomide in our study was 25 mg (QD d1‐21), and there was no significant difference in the number of induction courses. However, in the IMWG consensus, the early mobilization of stem cells is recommended, preferably within the first four cycles of lenalidomide therapy.^25^ A meta‐analysis including 1348 MM patients found that the initial use of lenalidomide (up to four cycles) was associated with CD34+ cell collection.^26^ The above results suggested that more than four courses of treatment with lenalidomide may have an effect on stem cell collection, but our results showed that more than three courses of RAD treatment had a significant effect on stem cell collection. In addition, with regard to the mobilization scheme for stem cell collection, among the patients in the RAD group who underwent stem cell mobilization in our study, 17 patients underwent CTX+G‐CSF mobilization, with a success rate (CD34+ cells ≥2 × 10^6^/kg) of 35.3%, while both of the patients mobilized by G‐CSF alone failed. Compared with that in previous studies, in the study by Baz, R C, the mobilization regimens included G‐CSF alone, G‐CSF+plerixafor, and G‐CSF+CTX, while CD34+ cells were collected from all patients with different mobilization regimens ≥2 × 10^6^/kg.^8^ It is undeniable that there are individual differences between China and Western countries, but the use of plerixafor in the mobilization process seems to offer hope for high‐risk patients with stem cell collection failure.^27^

Currently, with the continuous emergence of new drugs, the regimens for inducing NDMM are also constantly updated, such as the current international standard VRD regimen. A prospective phase III clinical trial enrolled 458 NDMM patients and reported that after six courses of VRD regimen, the CR rate was 33.4%, and the MRD negative rate was 28.8%, which was higher than that of the PAD or RAD regimen in our study. The incidence of grades 3–4 TEAEs was low, stem cell mobilization was carried out after three courses of VRD, and only 0.5% of the patients failed to collect enough CD34+ cells.^28^ Thus, in the next step, we can also consider combining bortezomib and lenalidomide to induce NDMM in patients because the combination of two different mechanisms of drug induction can increase the curative effect in principle. To avoid the adverse effect of lenalidomide on stem cell collection, we can also mobilize stem cells in advance on the basis of improving efficacy.

Our study has some limitations. Since this study was a nonrandomized controlled study, the relationship between treatment factors and outcomes was easily affected by confounders. Therefore, we used PSM to minimize bias and ensure similarities between the two groups. In addition, the number of patients was insufficient because in the course of the trial, we found that the RAD regimen affected stem cell collection and further affected ASCT, so the trial was terminated in time. However, insufficient sample size may lead to related negative results, but the obvious significant difference in stem cell collection between the two groups is worthy of deep consideration and continued research.

## 5 CONCLUSION

Compared to PAD induction, RAD induction had a significantly better safety profile, improved the quality of life of patients, and reduced occupational stress for doctors. The efficacy was comparable between RAD and PAD induction for patients with low‐risk NDMM. However, our research seems to indicate that the cycles of RAD induction should be limited to within four cycles to avoid irreversible damage to hematopoietic stem cells, but a larger sample size is still needed to verify the reliability of this conclusion.

## CONFLICT OF INTEREST

The authors have no financial interest to disclose in relation to the content of this article.

## ETHICAL APPROVAL

All procedures performed in studies involving human participants were in accordance with the ethical standards of the ethics committee of the first affiliated hospital of Sun Yat‐sen University ([2019]026) and with the 1964 Helsinki Declaration and its later amendments or comparable ethical standards. This article does not contain any studies with animals performed by any of the authors.

## INFORMED CONSENT

Informed consent was obtained from all individual participants included in the study.

## Supporting information

Fig S1‐S2‐Table S1‐S3

## Data Availability

The data supporting the findings of this study are available in the article and its Supplementary Materials.
