# Supplementary material for: Comparison of efficacy, safety, patients’ quality of life, and doctors’ occupational stress between lenalidomide‐based and bortezomib‐based induction in patients with newly diagnosed multiple myeloma
Source: Cancer Med. 2021 Feb 2;10(5):1656–67. doi: 10.1002/cam4.3762 (PMC7940229; doi:10.1002/cam4.3762)
Supplement: Supplementary file 1 — Fig S1‐S2‐Table S1‐S3 [file CAM4-10-1656-s001.docx]

**Supplementary information**

**1. Quality of life and occupational stress assessments**

The QLQ-C30 is a 30-item scale suitable for all tumor patients, with a total of 15 dimensions, including 5 functional dimensions, 3 symptom dimensions, 1 overall health status dimension and 6 single items. The MY20 is a scale designed for patients with MM that includes 2 functional dimensions and 2 symptom dimensions. According to the guidelines of the QLQ-C30 and MY20, the higher the score of the functional dimensions is, the better the health status is, and the higher the score of the symptom dimension is, the worse the quality of life is. To compare the effects of different treatment schemes on the quality of life of patients, we sent out questionnaires to evaluate the quality of life of patients in the RAD and PAD groups before treatment and after 2 and 4 courses of induction therapy. The numbers of valid questionnaires collected in the RAD group before treatment and after 2 and 4 courses of induction therapy were 28, 24, and 21, respectively, while those in the PAD group were 25, 22 and 21.

The questionnaire of effort-reward imbalance was used to evaluate doctors' and nurses' occupational stress, and the simplified Chinese version was developed by Dr. Jian Li. The questionnaire includes several questions we established, including whether there is worry about the side effects of drugs (often, sometimes, occasionally), whether there is worry about the average hospital stay of patients (yes, no), and the time it takes for the nurse to perform the treatment. The questionnaire consists of 23 questions. According to the score, we can obtain the pay/return ratio; the higher the ratio is, the greater the pressure. The higher the score of overcommitment is, the more intrinsic input.


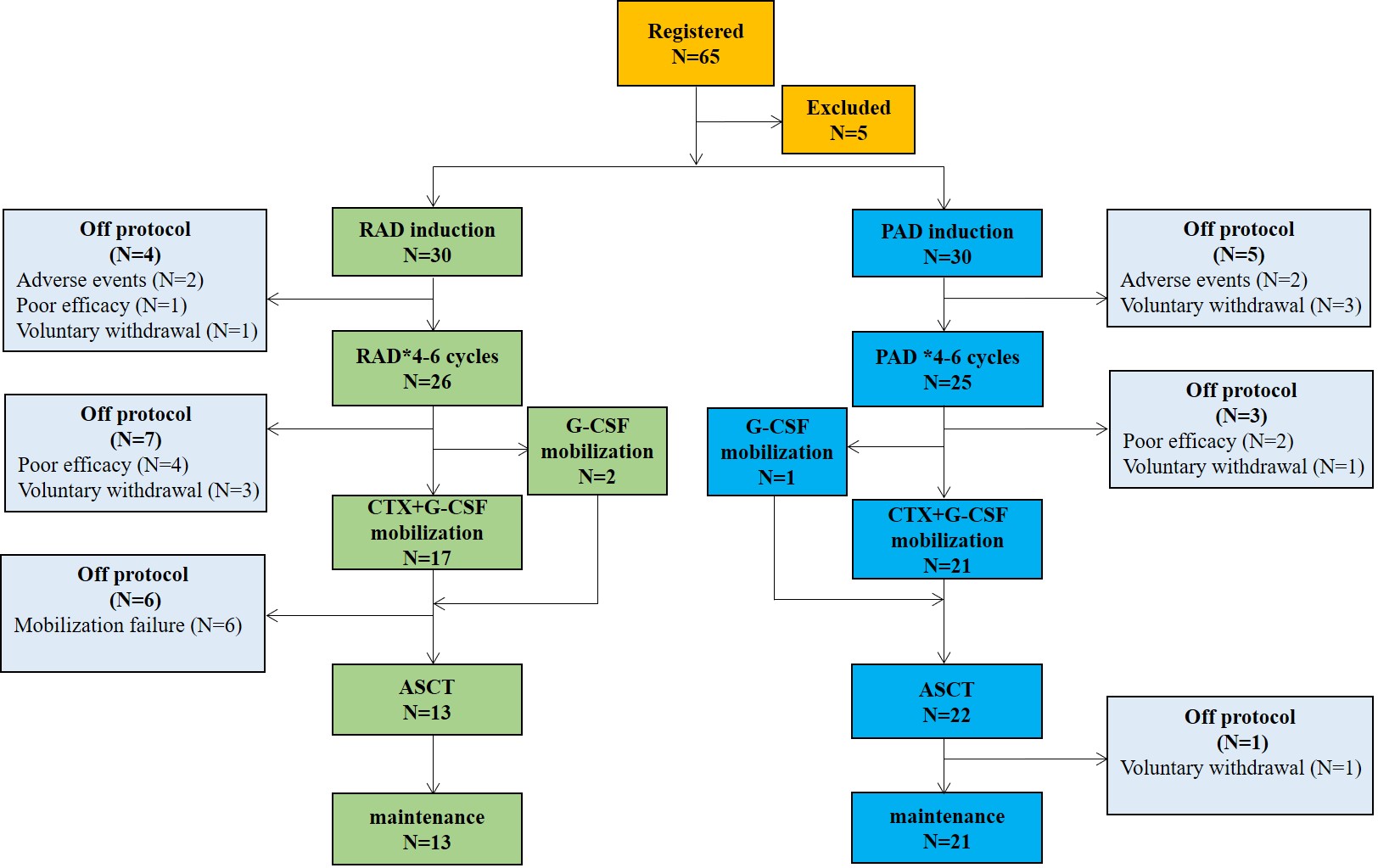
**Supplementary Fig. 1.** Consort diagram of patients.


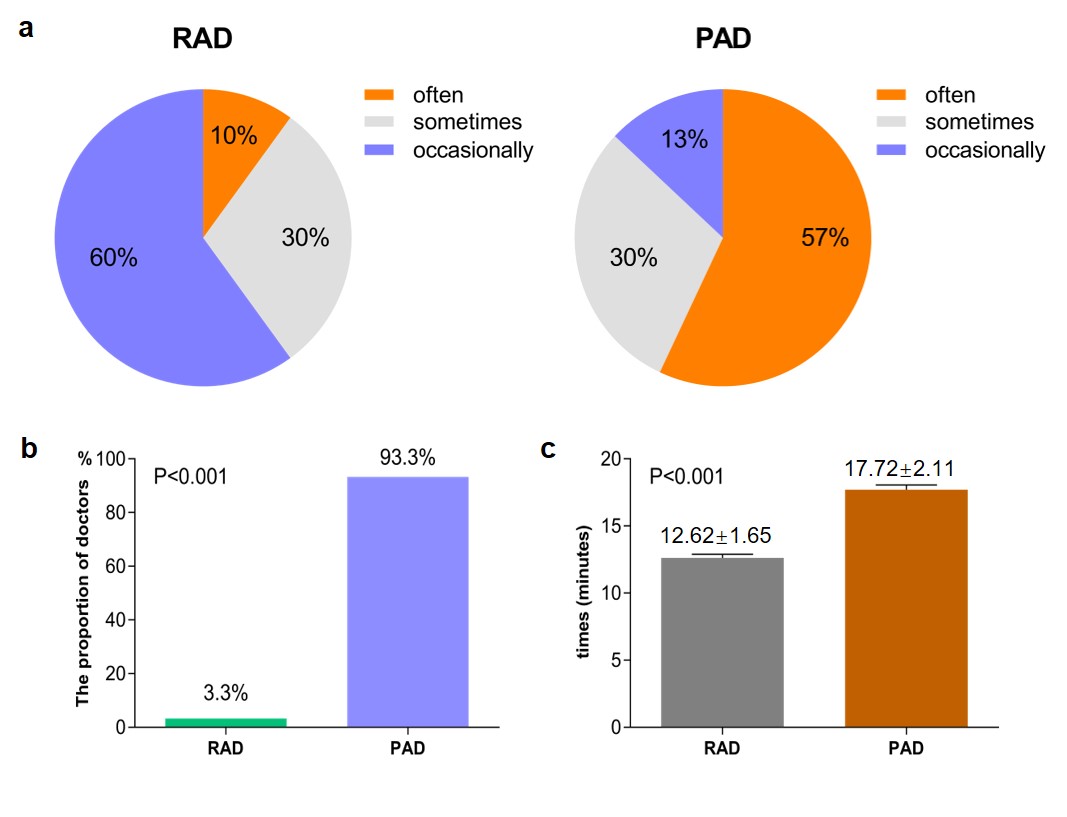
**Supplementary Fig. 2.** Comparison of the occupational stress of doctors and nurses using RAD vs. PAD.

(a) Comparison of the degree to which doctors are concerned about side effects with the RAD or PAD regimens. Sixty percent of doctors occasionally worry about the drug side effects of the RAD regimen, 30% of doctors sometimes worry about the drug side effects of the RAD regimen, and only 10% of doctors often worry about the drug side effects of the RAD regimen. However, for patients treated with the PAD regimen, only 13% of doctors occasionally worry about the drug side effects, and 57% of doctors often worry about the drug side effects, which was significantly different from that of using the RAD regimen (P<0.001).

(b) The proportion of doctors concerned about the problems caused by the average length of stay of patients in different groups. The use of the PAD scheme made more doctors worry about this problem than the RAD scheme (93.3% vs. 3.3%, P<0.001).

(c) Comparison of nurses' recording and processing time for different groups. Compared with the RAD regimen, it took more time for nurses to record and handle the PAD regimen (17.72 ±2.11 vs. 12.62 ±1.65, P<0.001).

**Supplementary Table 1.** Subgroup analysis of the CR rate at the end of induction chemotherapy in the two groups

| Complete response | Low-risk group | High-risk group | *P* |
| --- | --- | --- | --- |
| RAD (n=26) | 83.3% | 15.0% | 0.007 |
| PAD (n=25) | 50.0% | 26.3% | 0.560 |
| *P* | 0.540 | 0.633 |  |

**Supplementary Table 2.** Changes in the quality of life after induction therapy in the RAD group

| **Scales** | **RAD** | **Scores (mean ± SEM)** | ***P*** |
| --- | --- | --- | --- |
| **QLQ-C30 functional scales (physical)** | before treatment | 57.61±5.14 | <0.001 |
|  | 2 courses | 73.88±4.05 |  |
|  | 4 courses | 80.17±2.71 |  |
| **QLQ-C30 functional scales (role)** | before treatment | 56.54±5.77 | <0.001 |
|  | 2 courses | 80.55±5.08 |  |
|  | 4 courses | 83.33±3.63 |  |
| **QLQ-C30 symptom scales (pain)** | before treatment | 47.02±5.28 | <0.001 |
|  | 2 courses | 22.91±4.23 |  |
|  | 4 courses | 19.04±3.31 |  |
| **QLQ-C30 global health status** | before treatment | 38.69±4.50 | <0.001 |
|  | 2 courses | 63.19±3.68 |  |
|  | 4 courses | 76.98±2.43 |  |
| **MY20 disease symptoms** | before treatment | 43.60±2.51 | <0.001 |
|  | 2 courses | 23.39±3.90 |  |
|  | 4 courses | 18.74±3.87 |  |
| *QLQ-C30* quality of life questionnaire-C30; *MY20* myeloma-specific module 20. | | | |

**Supplementary Table 3.** Changes in the quality of life after induction therapy in the PAD group

| **Scales** | **RAD** | **Scores (mean ± SEM)** | ***P*** |
| --- | --- | --- | --- |
| **QLQ-C30 functional scales (physical)** | **before treatment** | 58.40±5.25 | 0.009 |
|  | **2 courses** | 72.42±3.11 |  |
|  | **4 courses** | 77.46±4.51 |  |
| **QLQ-C30 functional scales (role)** | **before treatment** | 45.33±5.73 | <0.001 |
|  | **2 courses** | 64.39±4.43 |  |
|  | **4 courses** | 80.95±4.78 |  |
| **QLQ-C30 symptom scales (pain)** | **before treatment** | 42.00±6.87 | 0.024 |
|  | **2 courses** | 29.54±4.90 |  |
|  | **4 courses** | 19.84±4.08 |  |
| **QLQ-C30 global health status** | **before treatment** | 37.66±4.14 | <0.001 |
|  | **2 courses** | 51.89±3.05 |  |
|  | **4 courses** | 68.25±3.52 |  |
| **MY20 disease symptoms** | **before treatment** | 42.19±3.75 | 0.031 |
|  | **2 courses** | 31.45±3.61 |  |
|  | **4 courses** | 27.62±4.72 |  |
| *QLQ-C30* quality of life questionnaire-C30; *MY20* myeloma-specific module 20. | | | |
